# Active assessment of fitness and performance in a general population

**DOI:** 10.3389/fspor.2025.1552365

**Published:** 2025-07-02

**Authors:** Brent Winslow, Aravind Natarajan, Samantha Mravca, Justin Julian Duong, Salahuddin Choudhary, Tracy Norman Giest

**Affiliations:** Google Research, Mountain View, CA, United States

**Keywords:** balance, exercise test, flexibility, muscle strength, physical fitness

## Abstract

Much of the current worldwide morbidity results largely from unhealthy diet, inactivity, and inadequate access to health resources. Improving body composition, strength, endurance, and cardiorespiratory fitness is associated with increased longevity and function, but current fitness assessments are largely qualitative and episodic. The current study sought to examine the feasibility of various bodyweight exercises in a general population, compare exercise performance metrics to reference measures, and develop a comprehensive fitness and performance assessment battery tied to longevity metrics. A group of adult research subjects (*n* = 152) from a convenience sample performed a series of 13 exercises and reference tests across balance, strength, endurance, flexibility, and cardiorespiratory fitness. While the majority of participants could perform all exercises, sex and age-related differences were observed in exercise performance. Isotonic exercises, such as push ups or floor triceps dips correlated more closely with reference measures than isometric exercises, such as squat and plank holds, which were associated with ceiling effects. Using this data, a comprehensive active assessment is proposed to screen for changes to fitness and provide individualized recommendations.

## Introduction

The leading causes of global death and disability are non-communicable diseases and include heart disease, stroke, chronic obstructive pulmonary disease (COPD), and diabetes, among others (1). While significant differences in mortality and disability have been observed across regions, ages, sexes, and income groups, much of the current worldwide morbidity is considered preventable, and results largely from unhealthy diet, inactivity, and inadequate access to health resources (2). In an effort to improve longevity and healthspan, available evidence has shown the prognostic value of measuring and tracking various factors across body composition (3), balance (4), upper and lower body strength (5, 6), core endurance (7), and cardiorespiratory fitness (8–10). The ability to measure and monitor changes to such factors could promote improved health, mobility, muscle strength, endurance, flexibility and posture, which are associated with increased longevity, healthspan, and functional capabilities (11, 12).

While current clinical approaches to longevity and healthspan often rely on individuals seeking care after symptoms arise, a number of approaches have sought to identify early indicators of health factors and provide targeted interventions to improve functional outcomes. Simple, self-report approaches (13) have been helpful, but are not comprehensive, have low resolution, and do not provide individualized recommendations. At-home fitness assessments (14) provide guidance for assessing aspects of body composition, balance, upper body function, lower body function, core endurance, cardiorespiratory fitness (CRF), and flexibility but remain largely qualitative and episodic. While physical test batteries have been proposed for specialized populations (15–17), what is needed is a comprehensive test battery that can be performed by a large portion of the adult population, without specialized equipment, and which provides quantitative, individualized results tied to health factors.

The purpose of this study was to examine the feasibility of a series of simple, bodyweight exercises targeting functional balance, upper body, core, and lower body strength and endurance, along with CRF and flexibility in a general population, and correlate the performance of these candidate exercises with established reference measures. Additionally, a series of novel, simplified, equipment free measures of CRF were compared to standard measures to determine feasibility and correlational metrics. Finally, the development of a comprehensive assessment battery using bodyweight exercises for a general population is described.

## Methods

### Subjects

All methods involving research subjects were approved by an Institutional Review Board (Advarra, Columbia, Maryland). Adults (aged 18–59) who were Google employees in the San Francisco, California Bay Area were recruited to participate in the study, which lasted approximately 60–90 min. Subjects were recruited using internal emails and chat spaces. Inclusion criteria included a lack of existing conditions such as diagnosed heart conditions, high blood pressure, angina, loss of balance or consciousness, chronic medical conditions, bone, joint, or soft tissue problems made worse by physical activity, or a need for medical supervision during physical activity that would prevent the subject from participating as assessed using the Physical Activities Readiness Questionnaire (PAR-Q) (18).

### Testing procedures

Upon arrival, subjects provided written, informed consent and were introduced to the testing protocol. Height and weight measurements were taken using a high precision scale (Seca 763, Hamburg, Germany), and an ECG strap was placed on each subject (Polar H10, Worcester Massachusetts). Prior to starting exercises, subjects provided a 5 min seated pre-exercise heart rate. Subjects were then randomized to one of three exercise orders via block randomization. During this time, instrumented reference measures and body-weight exercises were completed for functional balance, upper body capacity, core capacity, lower body capacity, cardiorespiratory fitness, flexibility, along with rest periods. Reference measures were included for each category that were well-established in the field, provided quantitative results, had normative data available for comparison, and were considered safe and accessible to an adult population cleared to exercise. Bodyweight exercises were also included for each category based on their ability to be performed without specialized equipment or spaces, were considered safe and accessible to an adult population cleared to exercise, and were capable of being part of an assessment battery that is comprehensive, quantitative, and associated with health factors. Subjects were provided encouragement and verbal feedback throughout all exercises. Following completion of all exercises, which took approximately 90 min, the ECG strap and watch were removed, and subjects were debriefed. A subset of subjects were invited to return on another day and undergo maximal testing for VO2max, which took an additional 30 min.

### Functional balance

#### Three stage balance test (reference measure)

The first three stages of the four stage balance test were used to assess static balance and measure the ability to hold a series of increasingly challenging balance positions, for at least 10 s each (19). Subjects were instructed in each of the positions, and held each position for 10 s, including a parallel stance, semi-tandem stance (performed once with the dominant foot forward, and once with the non-dominant foot forward), and tandem stance (performed once with the dominant foot forward, and once with the non-dominant foot forward). Subjects were given 5 s between each stance to switch to the next position. Throughout the test, subjects removed their shoes, placed their hands on their hips, and centered their balance between the two feet. The exercise was scored as the total number of seconds held across positions, up to a maximum of 50 s.

#### One legged-stance (OLS; bodyweight exercise)

Following the three stage balance test, subjects immediately started the 1 min one-legged stance (OLS) by raising the non-dominant foot, resting the dorsal part of the foot on the back of the opposite lower leg, and holding the position as naturally as possible (4). Subjects placed their hands on their hips, and fixed their gaze on a point at eye level, and held the position for up to 60 s. The sequence ended if: (1) the subject removed their hands from their hips; (2) shifted the standing foot (e.g., foot rotation to maintain balance); (3) shifted the non-dominant foot; (4) or had any other large body shift. The exercise was scored as the number of seconds the subject was able to hold the stance (20).

### Upper body capacity

#### Grip strength (reference measure)

To measure maximal handgrip strength, a Jamar hand dynamometer (APi Group, New Brighton, Minnesota), was used and set to the second handle position. Subjects were seated with their knees and elbows at 90 degrees, without using armrests. Any rings or jewelry that interfered with the dynamometer were removed. Subjects were instructed to squeeze the dynamometer as hard as possible with their right hand for 3 s, followed by a rest for at least 15 s, repeated three times. The test was then repeated with the left hand (21).

#### Push ups (bodyweight exercise)

Male subjects performed standard push ups, and female subjects had the option of performing either standard or modified push ups. Standard push ups were performed by assuming a plank position, with the fingers pointing forward, arms shoulder-width apart, back straight, head up, and using the toes as the pivot point. Modified push ups were performed in a plank position with the knees as the pivot point, lower legs in contact with the mat with ankles plantar flexed. Subjects then raised the body by fully straightening the elbows, and returned to the down position, lowering the body until the elbows were at least 90 degrees and the chest was within 2 inches of the floor, and continued for up to 1 min (22).

#### Floor triceps dips (bodyweight exercise)

Floor triceps dips were performed by having the subject sit with knees at 90 degrees, heels touching the floor, hands shoulder width apart, and fingers pointing toward the feet. Then, the subject extended their arms and pressed through their heels to lift the lower body from the ground. Each repetition was performed by bending the elbows and lowering the body without the glutes touching the floor, then returning to the starting position for up to 1 min.

### Lower body capacity

#### Knee extension strength (reference measure)

Portable fixed dynamometry was used to assess knee extension strength (23). A force meter (FC1k, Torbal, Bohemia, New York, USA) was fixed to a stable, high chair fitted with a seatbelt. The subject's dominant leg was connected to the force meter using an ankle cuff for cable machine attachment immediately above the malleolus, perpendicular to the tibial crest. The cable length was then adjusted so the subject's knee was held at 90 degrees. Subjects then performed a maximal isometric knee extension for 3 s, repeated 3 times, with at least 30 s between each test, with the maximal force recorded (24).

#### Squat hold (bodyweight exercise)

The squat hold was performed by standing with legs shoulder width apart, toes pointed forward, then lowering into a squat position with the knees flexed, and thighs parallel with the floor. Hands were clasped or held in front of the body to maintain balance, and the position was held for up to 1 min. The maximum duration in which the squat position was maintained was recorded.

### Core capacity

#### Abdominal crunches (reference measure)

Abdominal crunches were performed by lying flat on the back with feet on the floor, and knees held shoulder width apart. The head and neck were held in a neutral position. Hands were placed at the sides of the head, and the torso was lifted up while contracting the abdominal muscles, until the shoulders left the floor, followed by a relaxation of the abdominal muscles and a return to the floor, repeated for up to 1 min.

#### Plank hold (bodyweight exercise)

The plank hold (prone bridge test) is an isometric core exercise in which the body is held off the ground, face down, and supported by the forearms and toes. The plank hold was performed by placing the forearms on the floor, arms shoulder-width apart, palms flat, shoulders at 90 degrees, back straight, toes flexed, with the body held in a rigid plank. The heels, hips, and shoulders formed a straight line without arching the back or lifting the glutes. This position was held for as long as possible, up to 2 min.

### Cardiorespiratory fitness

#### Step test (reference measure)

Prior to performing the step test, subjects rested until their heart rate was within 10% of the pre-exercise heart rate. The step test was performed by having subjects repeatedly step onto and off of a 12 inch step, at a rate of 96 steps per min aided by a metronome. Subjects continue stepping for 3 min, then sit down on the step for a 1 min recovery. Heart rate data was then used to estimate VO_2_max using the heart rate recovery along with demographic data such as sex, age, height, and weight (25, 26).

#### VO_2_max via indirect calorimetry (reference measure)

A subset of subjects (10%) performed a modified Bruce protocol in conjunction with indirect calorimetry using a metabolic cart (ParvoMedics TrueOne 2400, Salt Lake City, Utah), to directly assess VO_2_max (27). First, the subject was weighed, fitted with a ECG strap (Polar H10) along with a mask that was adjusted until it was airtight. The subject was introduced to the protocol, along with exit criteria, which included a reported Borg RPE of 18 or above, a heart rate nearing the age-adjusted maximum (28), and a respiratory exchange ratio (RER) of approximately 1.1. The subject then chose an appropriate walking and running speed with the experimenter. Once the protocol started, the subject provided a Borg RPE value each minute, and the RER, heart rate, and stage were recorded. The protocol includes 1 min of walking at a flat incline, followed by 3 min of running at a flat incline. Following these initial stages, the running speed was held constant, and incline on the treadmill was increased to 1% for 2 min, then by an additional 2% every 2 min to a maximum of 9%. The maximal volume of oxygen used in ml/kg*min was then recorded. After reaching VO_2_max, the treadmill was returned to a flat incline, and the subject resumed moving at a walking speed to cool down for up to 5 min.

#### Run in place (bodyweight exercise)

Subjects were given the choice of performing either running in place or burpees for assessment of cardiorespiratory fitness. Prior to performing either test, subjects were required to rest until their heart rate was within 10% of their pre-exercise heart rate. For the run in place (stationary running) activity, subjects ran in place for 3 min, lifting their foot up to a mid-calf position with each step, to the beat of a metronome set to 160 beats per min to match the load of the step test. Following 3 min of running in place, subjects sit down for a 1 min seated recovery (29, 30).

#### Burpees (bodyweight exercise)

For the burpees activity, subjects repeatedly completed the 6 step gentle burpee: starting from a standing position, (1) subjects dropped into a supported squat, (2) stepped the left foot, (3) then the right foot into a plank, (4) then stepped the right foot into a supported squat, (5) followed by the left foot, (6) and finally stood up. Each step was done to the sound of a metronome set to 70 beats per min to match the load of the step test. Subjects continued performing the burpee protocol for 3 min, followed by a 1 min seated recovery.

### Flexibility

#### Sit and reach (reference measure)

To perform the instrumented sit and reach test, subjects removed their shoes and sat on the floor with their feet directly against the sit and reach measurement box. While keeping the legs fully extended and the knees straight, subjects placed one hand on top of the other, and while exhaling, leaned the body forward while pushing the slider as far as possible. Subjects held the stretch for 3 s, then returned to the starting position. The sit and reach test was repeated three times, and the best score in cm was recorded (31).

#### Stand and reach (bodyweight exercise)

To perform the stand and reach test, subjects stood tall with the knees fully extended and feet together. Without bending the knees, subjects then reached downward as far as possible (32). If the subject could touch the floor, they were instructed to reach farther and touch their knuckles to the floor. If the subject could reach their knuckles to the floor, they were instructed to try to place their palms on the floor. Reaches that could not touch toes were scored as 0, fingertip touches to the floor were scored as 1, knuckle touches to the floor were scored as 2, and palm touches to the floor were scored as 3.

### Statistical analyses

All statistical analyses were performed in Python using the Pandas, Matplotlib, Numpy, Scipy, and Seaborn libraries. Shapiro–Wilks tests were performed on the data to determine normality, followed by two-tailed, paired samples *t* tests with Bonferroni corrections to determine differences within subjects (e.g., reference vs. body weight measures), and two-tailed, independent samples *t* tests with Bonferroni corrections to determine differences between groups (e.g., sexes, age groups). Cohen's d effect size calculation, and *p* value computation on correlation coefficients was performed to measure the degree to which the data are consistent with a hypothesis that there is no trend in the population; *p* values less than or equal to 0.05 were considered significant.

## Results

The study was performed using a convenience sample of *N* = 152 subjects. All subjects were physically able to exercise based on responses to the PAR-Q. The average age of the subjects was 37.4 ± 10.7 (SD) years (range = 21–59 years), and the average BMI was 24.6 ± 3.73 (SD) kg/m^2^; 81 subjects were male and 71 were female (Table 1).

**Table 1:** Sociodemographic characteristics of the study sample.

Attribute	Study sample (%)
Gender	
Male	81 (53%)
Female	71 (47%)
Age Group	
20–29	48 (32%)
30–39	39 (26%)
40–49	34 (22%)
50–59	31 (20%)

Reference test performance is summarized in Table 2. All subjects were able to perform each reference test with the exception of the sit and reach exercise; one subject with back pain was unable to perform the exercise.

**Table 2:** Reference test performance summary.

Age	Female			Male		
	*N*	Mean	SD	*N*	Mean	SD
Balance test (s)						
20–29	24	50.0	0.0	24	49.9	0.6
30–39	22	49.7	1.3	17	50.0	0.0
40–49	14	50.0	0.0	20	49.7	1.3
50–59	11	49.1	2.4	20	49.0	3.0
Grip strength (kg)						
20–29	24	32.7	5.0	24	51.2	7.6
30–39	22	32.8	6.8	17	52.6	11.8
40–49	14	31.6	3.8	20	50.4	6.3
50–59	11	31.6	5.0	20	47.8	7.1
Knee extension strength (kgf)						
20–29	24	44.2	10.7	24	70.4	13.1
30–39	22	46.4	14.9	17	61.1	17.1
40–49	14	40.0	14.3	20	55.3	9.8
50–59	11	39.9	10.4	20	51.4	10.7
1 min crunches (reps)						
20–29	24	47.3	9.8	24	41.9	13.9
30–39	22	42.5	11.3	17	48.1	15.9
40–49	14	36.9	10.3	20	42.3	14.7
50–59	11	33.5	7.6	20	39.5	11.9
VO_2_max (ml/kg*min; estimated from step test)						
20–29	24	35.8	2.6	24	44.9	2.3
30–39	22	33.0	2.4	17	41.9	2.1
40–49	14	27.5	2.4	20	37.9	2.6
50–59	11	26.6	4.8	20	34.6	2.0
Sit and reach (cm)						
20–29	24	35.1	9.8	24	25.0	7.8
30–39	21	34.9	12.0	17	27.5	7.0
40–49	14	35.8	10.3	20	29.4	10.2
50–59	11	30.3	12.1	20	21.8	7.3

*N* represents the ratio of subjects by demographic that were able to perform the exercise. SD, standard deviation. All subjects were able to complete all activities with the exception of one subject unable to complete the sit and reach, highlighted in yellow.

Bodyweight exercise performance is summarized in Table 3. The majority of subjects were able to perform all bodyweight exercises. The exercise that was most difficult to perform was push ups, with 8/152 subjects unable to perform any push up repetitions. Of the subjects who were unable to complete one push up repetition, 3 cited pain (palm, shoulder, neck), and the rest either could not maintain the plank position, or perform a repetition with the required range of motion. The majority of subjects were also able to perform the lower body exercises. One subject with back pain was unable to perform the squat exercise in this study. There were ceiling effects observed with the static posture exercises (one-legged stance, forearm plank, and squat hold) in which the majority of subjects completed the maximal exercise duration, limiting the ability to detect variations in performance. Each exercise will be discussed in more detail in the following sections.

**Table 3:** Exercise performance summary.

Age	Female			Male		
	*N*	Mean	SD	*N*	Mean	SD
1 min one-legged stance (s)						
20–29	24	56.9	9.8	24	43.9	20.7
30–39	22	57.5	11.7	17	54.6	13.4
40–49	14	52.4	17.7	20	52.7	14.2
50–59	11	56.7	10.9	20	37.3	25.2
1 min push ups (reps)						
20–29	22	20.8	11.0	24	26.9	13.0
30–39	20	20.2	11.2	17	27.8	12.5
40–49	13	19.1	11.5	18	25.2	14.2
50–59	11	18.5	12.7	19	20.9	12.1
1 min floor triceps dips (reps)						
20–29	24	38.2	16.6	24	43.5	16.3
30–39	22	35.6	16.2	17	45.5	20.8
40–49	14	37.6	15.5	20	44.7	23.5
50–59	10	35.0	23.5	20	48.6	17.6
1 min squat hold (s)						
20–29	24	56.0	9.1	24	58.5	4.6
30–39	21	56.0	13.9	17	58.7	4.2
40–49	14	57.6	4.8	20	58.1	6.3
50–59	11	52.4	13.9	20	59.9	0.3
2 min forearm plank (s)						
20–29	24	94.5	33.7	24	102.1	25.6
30–39	22	104.6	26.0	17	107.2	24.9
40–49	14	97.6	29.4	20	108.3	23.2
50–59	11	96.3	23.8	20	112.3	19.7
VO_2_max (ml/kg*min; estimated from run in place or burpee)						
20–29	24	35.9	2.1	24	44.7	1.9
30–39	22	32.9	2.3	17	42.0	2.3
40–49	14	28.5	1.5	20	38.1	2.6
50–59	11	26.3	3.3	20	28.5	1.5
Stand and reach (categorical; 0 = no touch; 1 = floor touch; 2 = knuckles; 3 = palms)						
20–29	24	1.71	1.27	24	0.75	0.79
30–39	21	2.05	1.25	17	1.05	1.03
40–49	14	2.00	1.18	20	1.20	1.20
50–59	11	1.45	1.21	20	0.40	0.75

*N* represents the number of subjects by demographic that were able to perform the exercise. SD, standard deviation. Exercises that were not performed by all subjects are highlighted in yellow.

### Functional balance

#### Balance test

The majority of subjects (145/152) were able to complete each portion of the balance test without difficulty. Subjects who were unable to complete the full balance test were more likely to be older (4/7 subjects with difficulty were in their 50 s). Given the high performance of the sample, the balance test was not used for further analysis.

#### One legged-stance

**Figure 1:**
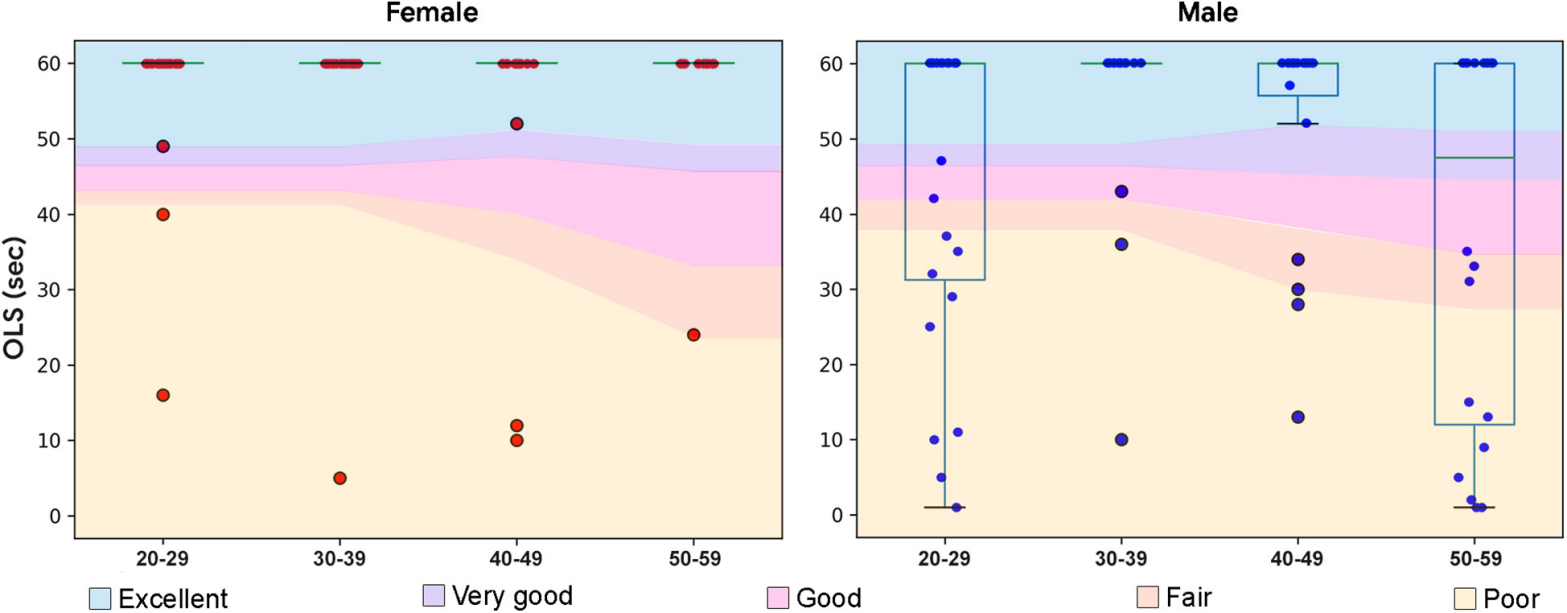
Boxplots overlaid with OLS performance data in seconds by demographic. Normative data from (33) with excellent represented as one standard deviation above the mean, very good as a half standard deviation above the mean, good as the mean, fair as a half standard deviation below the mean, and poor as a standard deviation below the mean.

The majority of subjects (76%), both males (63%) and females (89%) were able to hold the 1 min one legged-stance (OLS) (Figure 1). Female subjects held the stance significantly longer than male subjects (*p* < 0.001, *d* = 0.35), and performance was stable with age in this sample. Surprisingly, the 20–29 year old male subjects in this study demonstrated poor balance on this test, with only a 54% success rate.

### Upper body capacity

#### Grip strength

**Figure 2:**
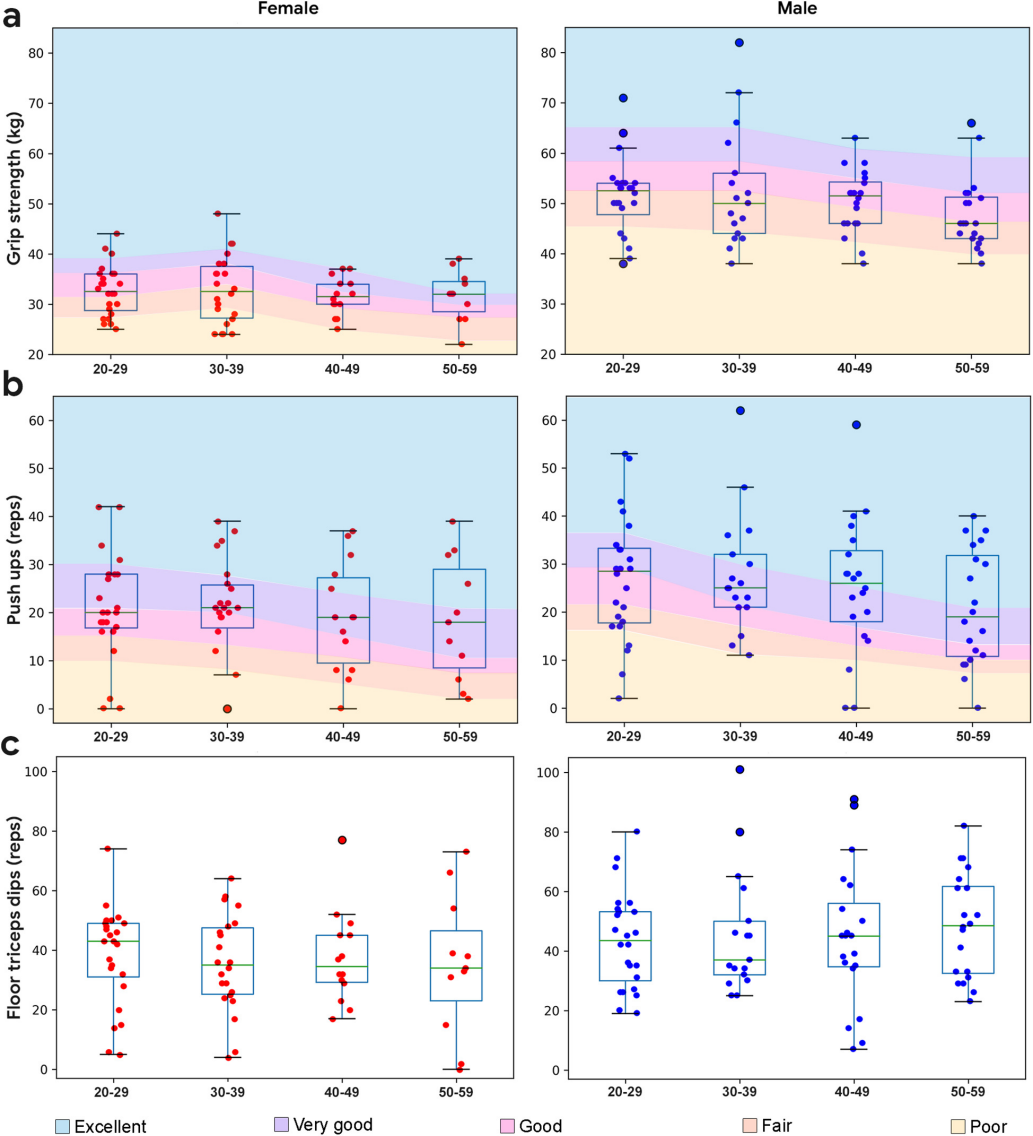
Boxplots overlaid with upper body performance data by demographic. **(a)** Dominant hand grip strength performance by demographic. Normative data from (34, 35). **(b)** Push up performance by demographic. Normative data from (36). **(c)** Floor triceps dips performance by demographic (no normative data available). Normative data has excellent represented as one standard deviation above the mean, very good as a half standard deviation above the mean, good as the mean, fair as a half standard deviation below the mean, and poor as a standard deviation below the mean.

Of the subjects in this study, 94% were right-handed and 6% were left-handed. Handgrip strength was captured using standard procedures (21). Dominant hand strength was higher than the non-dominant hand (*p* < 0.001, *d* = 0.10), and was higher in the male subjects than the female subjects (*p* < 0.001, *d* = 0.85), (Figure 2a). A decline in handgrip strength was observed with age, starting in the 4th decade for males and the 5th decade for females (37). Each demographic showed a wide range of values, with no ceiling effect. As captured, handgrip strength represents an appropriate reference metric for upper body capacity in this study sample.

#### Push ups

Push up performance is shown in Figure 2b. All male subjects were required to perform standard push ups. Similar to the grip strength data, a range of values was observed in each decade, with a decline in the median values starting in the 5th decade. Male subjects performed more push ups than female subjects (*p* = 0.0086, *d* = 0.27). Female subjects were given the option to perform modified (knee) or standard push ups. Modified push ups were preferred by the majority of subjects (83%), while the remainder chose standard push ups (14%) or were unable to perform this exercise (3%). Regardless of type of push up, the median performance for female subjects remained stable with age, similar to grip strength.

Push ups capacity (number of push ups performed per min) was significantly correlated with grip strength for the entire study sample (*r* = 0.52, *p* < 0.001), including male (*r* = 0.44, *p* < 0.001) and female (*r* = 0.28, *p* = 0.019) subjects.

#### Floor triceps dips

Floor triceps dips performance is shown in Figure 2c. Male subjects performed more floor triceps dips on average (*p* = 0.0041, *d* = 0.27), with similar median values observed at each decade, and a range of values observed. Subjects reported less confidence in performing the exercise due to its low familiarity.

Floor triceps dips capacity (number of floor triceps dips performed per min) was significantly correlated with grip strength for the entire study sample (*r* = 0.45, *p* < 0.001), including male (*r* = 0.37, *p* < 0.001) and female subjects (*r* = 0.24, *p* = 0.047).

### Lower body capacity

#### Knee extension strength

**Figure 3:**
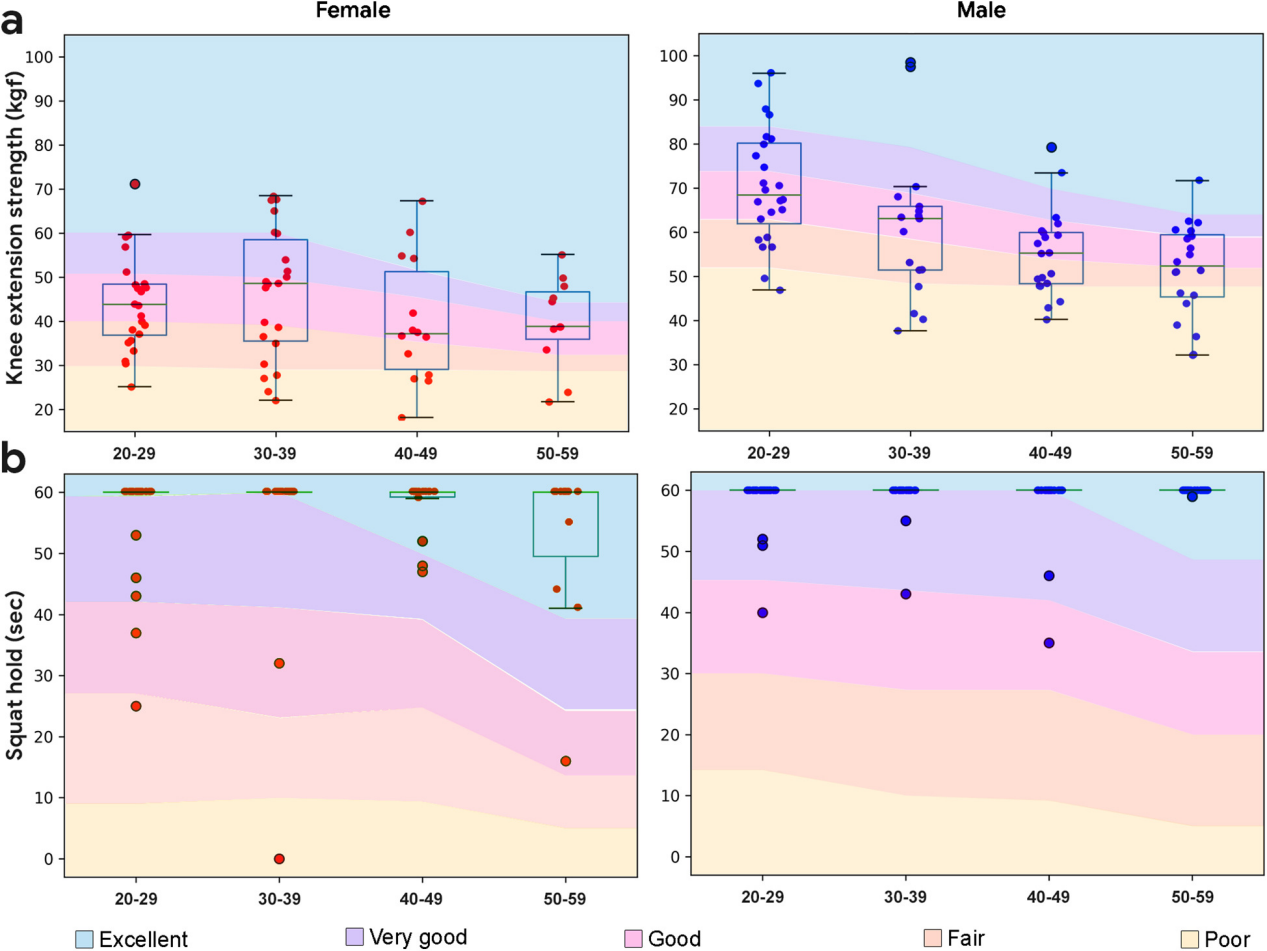
Boxplots overlaid with lower body performance data by demographic. **(a)** Knee extension strength performance by demographic. Normative data from (38–40). **(b)** Squat hold performance by demographic. Normative data from (41). Normative data has excellent represented as one standard deviation above the mean, very good as a half standard deviation above the mean, good as the mean, fair as a half standard deviation below the mean, and poor as a standard deviation below the mean.

Knee extension strength was quantified via portable fixed dynamometry using standard procedures (23) and is shown in Figure 3a. Knee extension strength was higher in the male subjects than the female subjects (*p* < 0.001, *d* = 0.54), and declined with age. Each demographic showed a range of values, with no ceiling effect. As captured, knee extension strength represents an appropriate reference metric for lower body capacity in this study sample.

#### Squat hold

One minute squat hold performance is shown in Figure 3b. Unlike knee extension strength, a clear ceiling effect was observed that prevented additional correlational analyses. The majority of subjects (85%), both males (89%) and females (80%) were able to hold the isokinetic squat for 1 min, and male subjects held the squat for significantly longer than female subjects (*p* = 0.024, *d* = 0.21).

### Core capacity

#### Abdominal crunches

**Figure 4:**
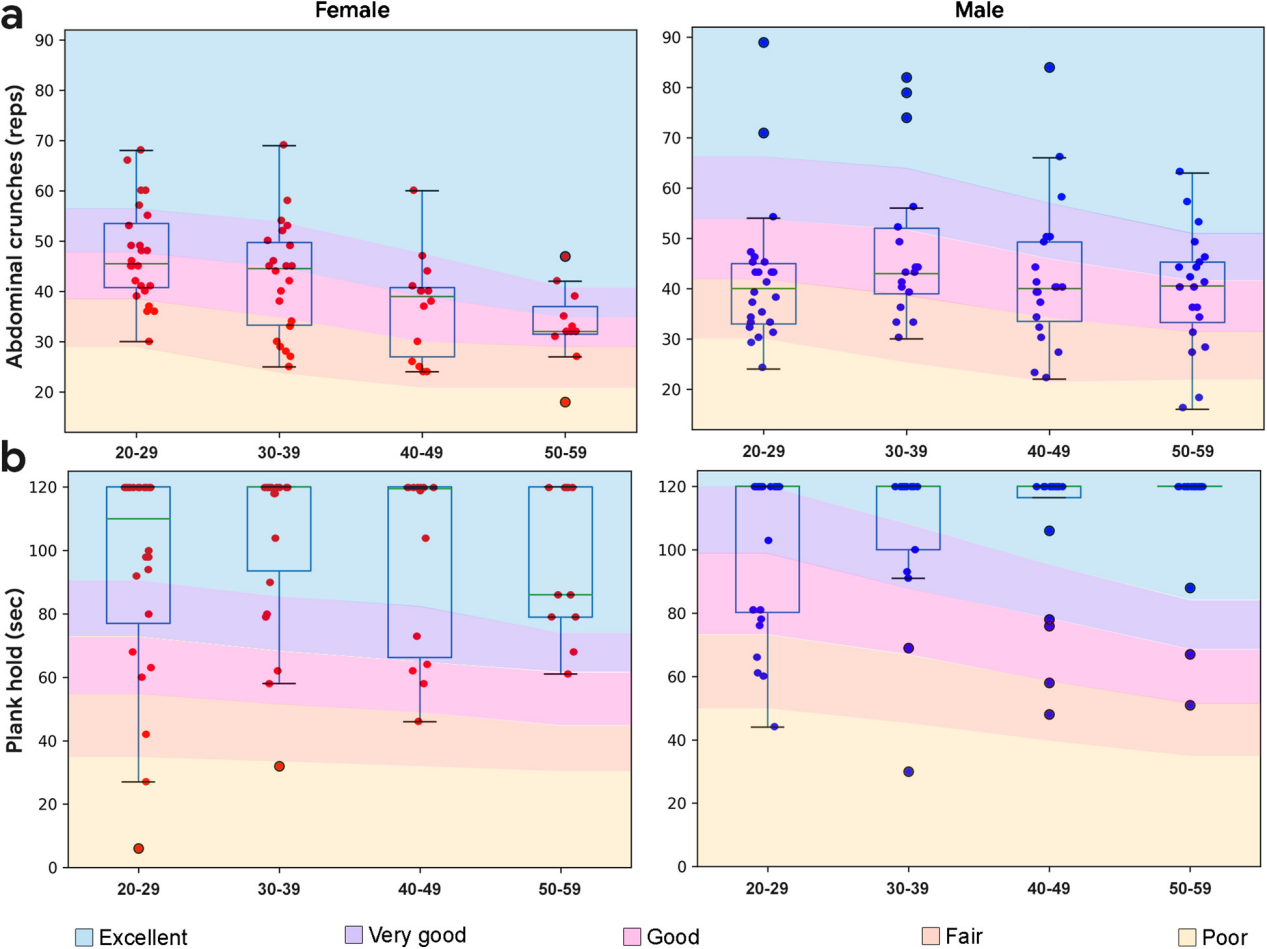
Boxplots overlaid with core body performance data by demographic. **(a)** Crunches performance by demographic. Normative data from (43). **(b)** Plank hold performance by demographic. Normative data from (20, 44). Normative data has excellent represented as one standard deviation above the mean, very good as a half standard deviation above the mean, good as the mean, fair as a half standard deviation below the mean, and poor as a standard deviation below the mean.

Crunches (or half sit-ups) represent a foundational supine to sitting movement for activities of daily living. Crunches were performed according to standard procedures (42). Maximum 1 min crunch performance is expected to decline with age and show minimal gender effects (42). In the study sample, crunch performance showed a range of values at each demographic, declined with age for female subjects (*r* = −0.46, *p* < 0.001) but not for male subjects (*r* = −0.09, *p* = 0.43), did not demonstrate a ceiling effect, and did not differ between the sexes (*p* = 0.61, *d* = 0.039; Figure 4a). As captured, 1 min crunches represent an appropriate reference metric for core capacity in this study sample.

#### Plank hold

Performance from the 2 min plank hold (or prone bridge test) is shown in Figure 4b. Unlike crunches, planks showed a clear ceiling effect which prevented additional correlation analyses. The majority of subjects (64%) were able to hold the plank for 2 min, including 59% of males and 73% of females. On average, male subjects held the plank longer than female subjects (*p* = 0.042, *d* = 0.19).

### Cardiorespiratory fitness

#### VO_2_max

A subset of subjects (*n* = 14) performed maximal testing using indirect calorimetry and a modified Bruce protocol on a treadmill. Male subjects (43.1 ± 3.45 ml/kg*min) had higher VO_2_max values than female (38.0 ± 5.77 ml/kg*min) subjects (*p* = 0.033, *d* = 1.08).

#### Step test

The 3 min step test was performed using the YMCA protocol (25). All subjects performed the entire step test, followed by 1 min of seated recovery. Heart rate during recovery along with demographic data (sex, age, height, weight) was then used to estimate VO_2_max (26):

(1) $$VO_{2}\max=c_{0}+c_{1}*\mathrm{sex}+c_{2}*\mathrm{age}+\;c_{3}*\mathrm{weight}+\;c_{4}*HRR$$

where the coefficients are (45) *c*_0_ = 84.5, *c*_1_ = –10.2, *c*_2_ = –0.4, *c*_3_ = –0.1, and *c*_4_ = –0.1.

**Figure 5:**
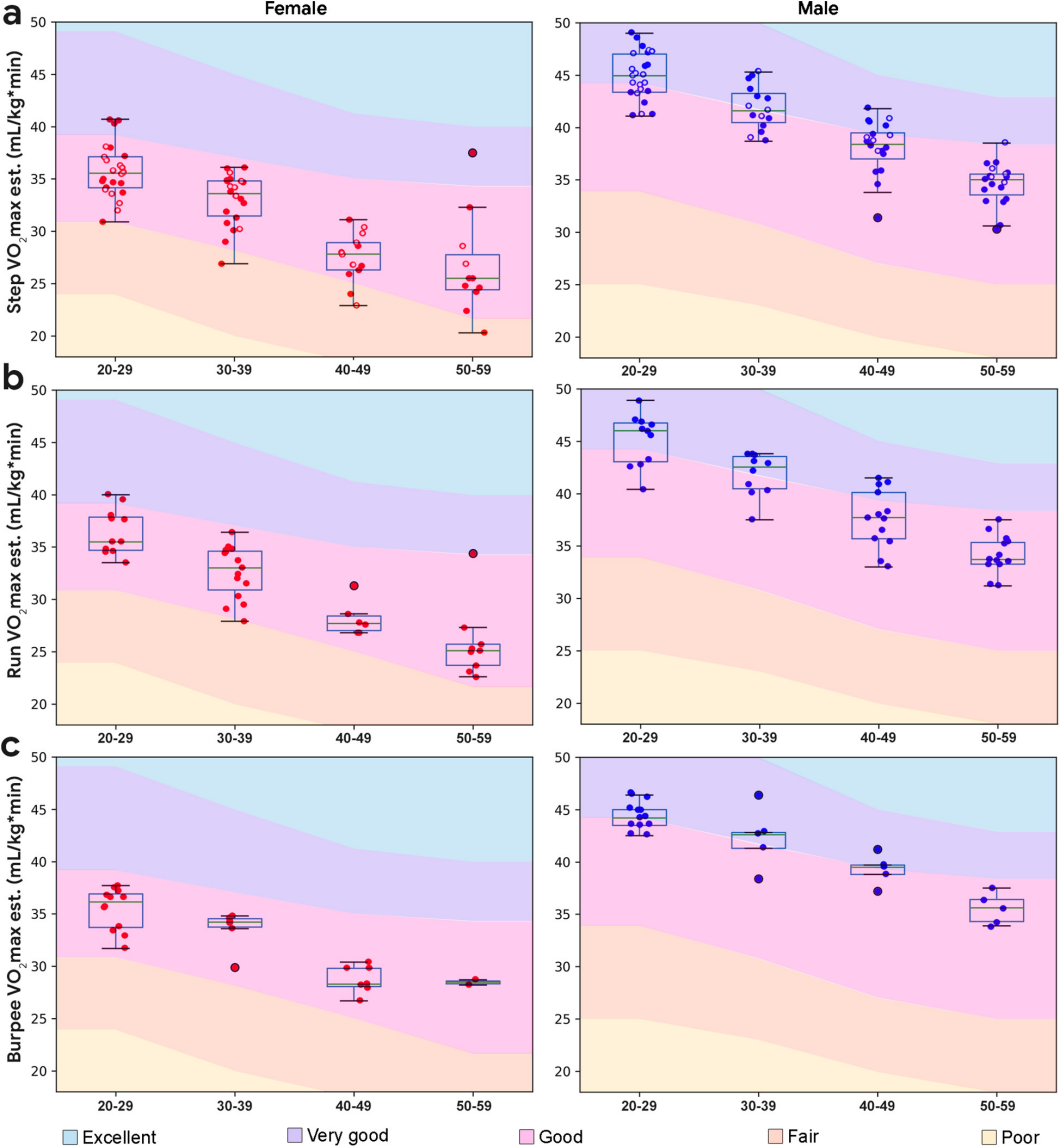
Boxplots overlaid with cardiorespiratory performance data by demographic. **(a)** Step test VO_2_max estimate by demographic; individuals who chose the run in place are plotted with filled circles, and individuals who chose the burpee test are plotted with unfilled circles. **(b)** Run in place VO_2_max estimate by demographic. **(c)** Burpees VO_2_max estimate by demographic. Normative data from (29).

Most subject's VO_2_max estimates were in the good to very good range, with scores from the male subjects exceeding those of the female subjects (*p* < 0.001, *d* = 1.73). A decline in VO_2_max estimates were observed with age for female (*r* = −0.77, *p* < 0.001) and male subjects (*r* = −0.88, *p* < 0.001) (Figure 5a). VO_2_max derived from the step test was correlated with VO_2_max measured using indirect calorimetry for the male (*r* = 0.83, *p* = 0.02, *n* = 7) and not the female subjects (*r* = 0.10, *p* = 0.83, *n* = 7).

#### Run in place

In addition to the step test, all subjects were given the option to perform a 3 min, paced run in place, or 3 min, paced gentle burpees, both followed by 1 min recovery. The majority of subjects (61%) chose to run in place, by a ratio that increased with age. The run in place exercise was reported to be more familiar by subjects.

Following (26), we modeled the estimation of VO_2_max from the run in place experiment, in a similar form (see Equation 1). Performing a linear regression using the VO_2_max derived from the step test data as the true labels, we obtain the following values for the coefficients: *c*_0_ = 84.506, *c*_1_ = –10.876, *c*_2_ = –0.415, *c*_3_ = –0.118, *c*_4_ = –0.068. With these coefficients, it is possible to estimate VO_2_max from the run in place test, using Equation 1. The mean absolute error averaged over 5 validation folds was found to be 1.24 ml/kg*min. Run in place performance is shown in Figure 5b. Similar to estimates derived from the step test, most subject's VO_2_max estimates were in the good to very good range, with scores from the male subjects generally exceeding those of the female subjects (*p* < 0.001, *d* = 1.59). VO_2_max derived from run in place was significantly correlated with VO_2_max measured using indirect calorimetry in a subset (*n* = 7) of the study sample (*r* = 0.75, *p* = 0.05).

#### Burpees

Burpee performance is shown in Figure 5c. Similar to the analysis performed with the run in place data, we modeled VO_2_max using Equation 1 and estimated the coefficients through linear regression. Similar to the run in place experiment, we used the VO_2_max values estimated from the Step Test as true labels. The values of the coefficients were found to be *c*_0_ = 80.419, *c*_1_ = −10.719, *c*_2_ = −0.365, *c*_3_ = −0.092, *c*_4_ = −0.058. These coefficients allow us to estimate VO_2_max using the burpees performance. The mean absolute error averaged over 5 validation folds was found to be 1.33 ml/kg*min. Similar to estimates derived from the step test and burpees, most subject's VO_2_max estimates were in the good to very good range, with scores from the male subjects generally exceeding those of the female subjects (*p* < 0.001, *d* = 2.5). Similar to the step test, a linear decline in VO_2_max estimates was observed with age for both run in place and burpee (*r* = −0.57, *p* < 0.001). Subjects reported that the 6 step gentle burpee exercise was more difficult and less familiar than running in place. VO_2_max derived from burpees was not significantly correlated with VO_2_max measured using indirect calorimetry in a subset (*n* = 7) of the study sample (*r* = 0.51, *p* = 0.30).

### Flexibility

#### Sit and reach

**Figure 6:**
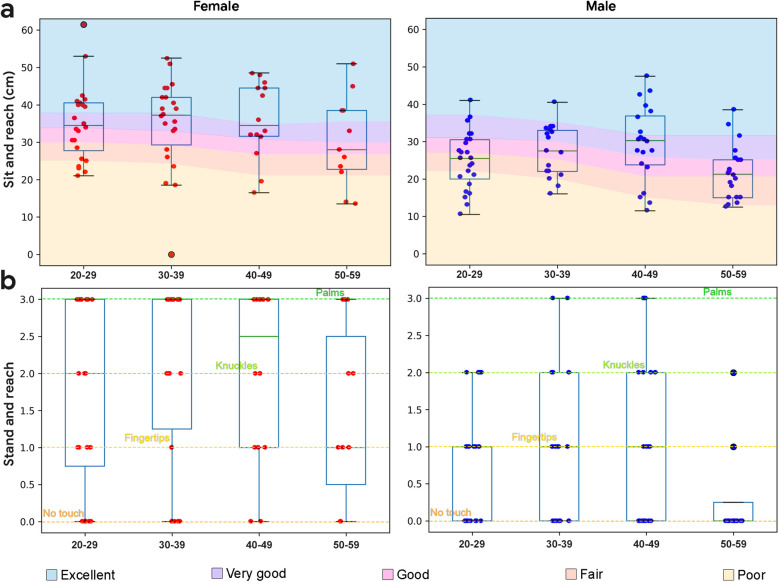
Boxplots overlaid with flexibility data by demographic. **(a)** Sit and reach performance by demographic. Normative data from (36). **(b)** Stand and reach performance by demographic (no normative data available).

Performance on the instrumented sit and reach test was done according to standard procedures (36), and presented in Figure 6a. Flexibility declined with age, especially in the 5th decade. Female subjects demonstrated higher flexibility than male subjects (*p* < 0.001*, d* = 0.56). The study sample also showed a range of values at each demographic, with no ceiling effect. As captured, the sit to reach test represents an appropriate reference metric for flexibility in the study sample.

#### Stand and reach

The stand and reach (or toe touch) test was used as an equipment free proxy for sit and reach. Rather than measure exact distances reached, categorization of reaches were used, including an inability to touch the floor, floor touches with fingertips, floor touches with knuckles, and floor touches with palms (Figure 6b). Categorical sit and reach performance (excellent, very good, good, fair, poor) was compared with categorical stand and reach performance. Female subjects demonstrated higher flexibility than male subjects (*p* < 0.001, *d* = 0.88).

Stand and reach performance was significantly correlated with sit and reach performance for the entire study sample (*r* = 0.83, *p* < 0.001), including both males (*r* = 0.76, *p* < 0.001) and females (*r* = 0.89, *p* < 0.001).

### Exercise performance correlations

#### Female subject correlations

**Figure 7:**
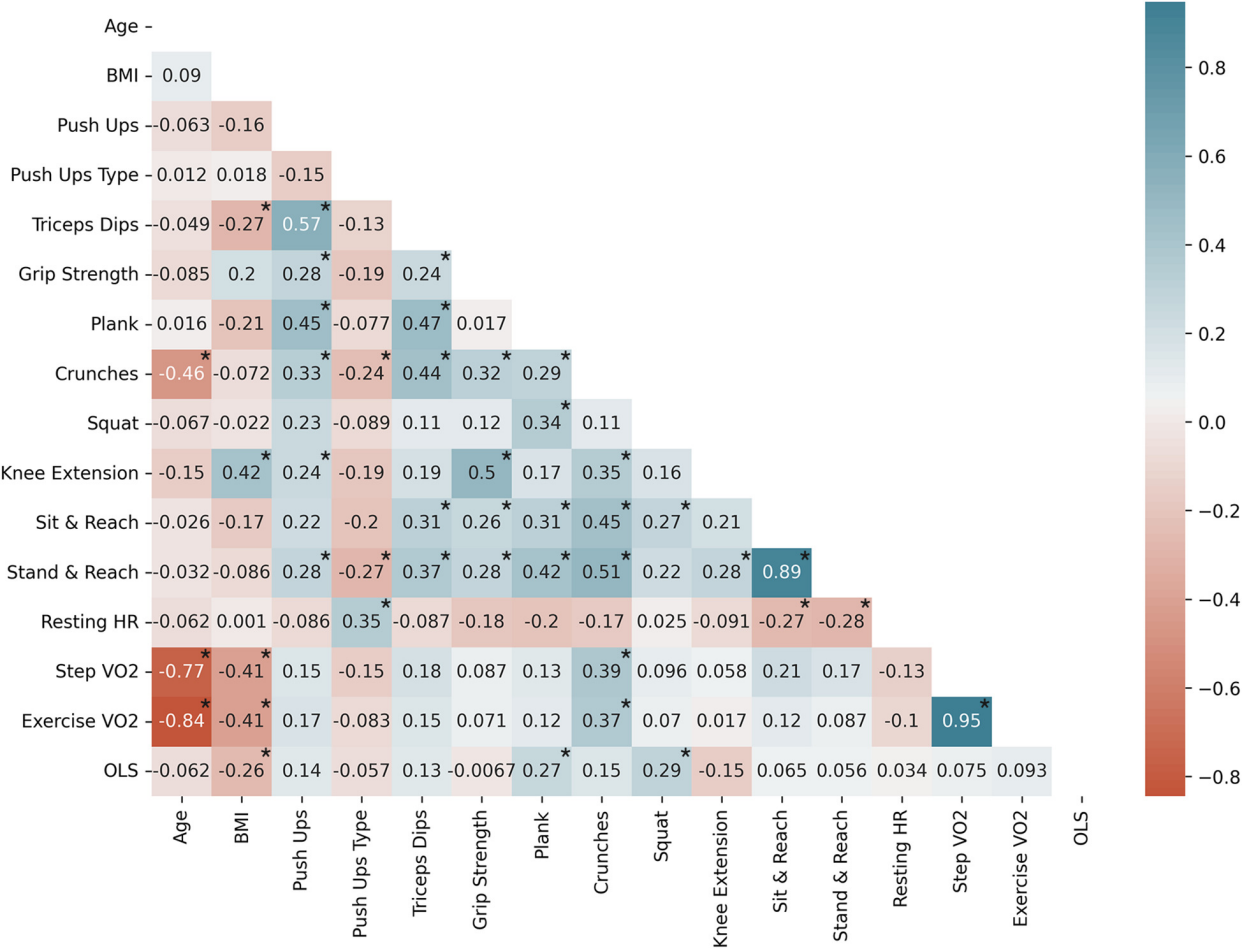
Female subject Pearson correlation coefficients (*n* = 71) by variable; significantly correlated variables are shown with*, *p* < 0.05. Exercise VO2 represents both run in place and burpee performance. Negative correlations are shown in red and positive correlations are shown in blue.

**Figure 8:**
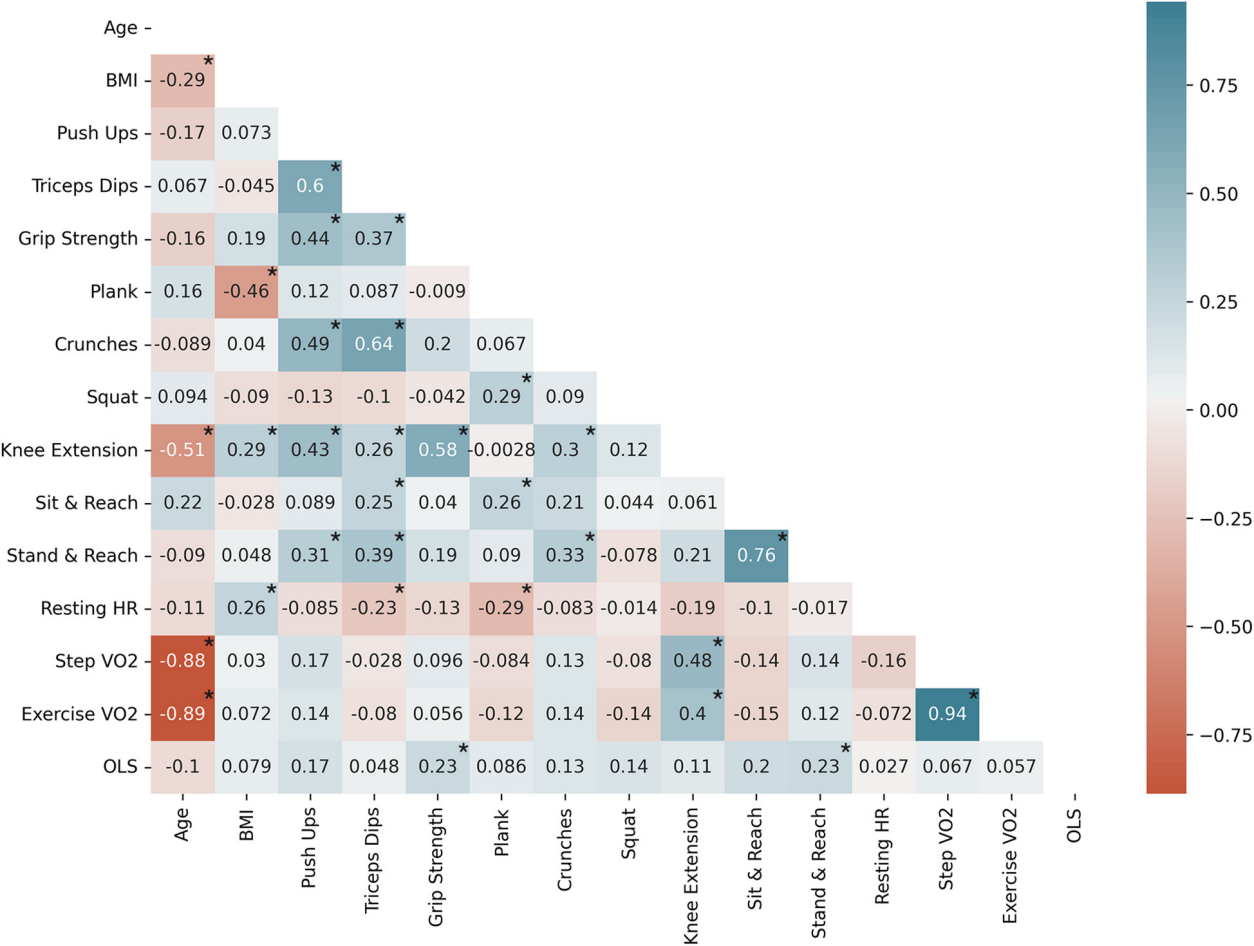
Male subject Pearson correlation coefficients (*n* = 81) by variable; significantly correlated variables are shown with*, *p* < 0.05. Exercise VO2 represents both run in place and burpee performance. Negative correlations are shown in red and positive correlations are shown in blue.

Due to the sex differences observed across exercises, correlations by sex are shown in Figures 7, 8. For female subjects (Figure 7), increasing age was negatively correlated with core capacity, an observation only seen in female subjects. Body composition was negatively correlated with cardiorespiratory fitness and balance; and grip strength was positively correlated with flexibility, observations only seen in female subjects. For female subjects, all isometric exercises (plank hold, squat hold, and OLS) were positively correlated.

#### Male subject correlations

For male subjects (Figure 8), increasing age was associated with a decrease in lower body capacity, a trend that was only observed in the male subjects. Upper body capacity was correlated with flexibility, and core capacity was associated with pre-exercise heart rate and derived VO_2_max. Increasing cardiorespiratory fitness was also correlated with lower body capacity.

An analysis of order effects was performed to support temporal sequencing of a recommended assessment. All orders showed low correlation to exercise performance, and none were statistically significant (data not shown).

### Assessment battery

Based on the results from this study, there is an opportunity to combine a series of bodyweight exercises into an assessment that is simple, fast, quantitative, and correlated with reference measures linked to health. The following exercises represent such an assessment: stand and reach; 1 min OLS; heart rate recovery following 3 min run in place; 1 min push ups; 1 min crunches; and 1 min squat reps. Immediately prior to starting the active assessment, subjects should warm up by performing simple exercises such as stretching or walking that do not generate fatigue (45). Next, stand and reach provides information about individual flexibility in a simple, rapid assessment, and can serve to provide additional warm up prior to upcoming cardiovascular and lower body capacity activities. Then, the 1 min OLS would be performed due to the potential for fatigue from the cardiovascular and/or lower body activities affecting balance performance. To date, many studies of the OLS only assess the dominant leg, but assessment of both legs may provide valuable information on asymmetries that could be improved via balance exercises. Following these initial warm up exercises, subjects would run in place for 3 min, followed by 1 min of seated recovery. Running in place is a highly familiar movement, and does not fatigue the upper body or core, unlike burpees. Performing cardiovascular exercise prior to strength/endurance exercises also increases blood flow throughout the body providing additional warmup to allow for recovery assessments prior to subsequent exercises which may fatigue major muscle groups. After cooling down, subjects would perform up to 1 min of push ups. Both standard and modified push ups should be supported in such an assessment. Next, subjects would perform up to 1 min of crunches. Unlike the plank hold, crunches did not produce a ceiling effect, and provide real-world relevance, such as moving from a supine to seated position. Crunches should be performed after push ups due to the potential for crunches to affect an individual's ability to hold a plank position during the push up. Finally, subjects would perform up to 1 min of squat repetitions, which, similar to crunches, has real-world relevance, such as moving from a seated to a standing position. Such a protocol could be accomplished in less than 10 min, and represents a more quantitative, actionable, and comprehensive assessment than available approaches (see Table 4).

**Table 4:** Existing comprehensive movement assessments.

Assessment	Balance	Upper body capacity	Lower body capacity	Core capacity	CRF	Flexibility
Current effort (order)	OLS (2)	1 min push ups (4)	1 min air squats (6)	1 min crunches (5)	HRR following 1 min run in place (3)	Stand and reach (1)
Mayo Clinic (46)	—	1 min push ups	—	1 min crunches	1.5 mi run	Sit and reach
Select Health (47)	OLS	1 min push ups	Wall sit	1 min plank	1 min jumping jacks	—
MSU (47, 48)	—	1 min push ups	—	1 min sit ups	HRR following 1 mile walk	Sit and reach
WFA (49)	OLS	1 min push ups	1 min air squats	Forearm plank	HRR following 3 min step test	Sit and reach
ACSM (36)	BESS; Y-balance test	1 rep max bench press; push ups	1 rep max leg press; vertical jump test	-	Step test; VO_2_max	Range of motion test by joint

BESS, balance error scoring system; CRF, cardiorespiratory fitness; HRR, heart rate recovery; MSU, Michigan state university; OLS, one-legged stance; WFA, wellnessed fitness assessment.

## Discussion

The primary goals of this study included assessing the feasibility of bodyweight exercises for fitness assessment, correlating these exercises with reference measures, evaluating simplified CRF measures, and developing a comprehensive assessment battery, were largely achieved. The results suggest that the chosen exercises were accessible to the majority of the study population, and significant correlations were observed between candidate exercises and established reference measures. Moreover, the novel CRF measures showed promising results, allowing for the proposal of a practical bodyweight assessment battery for a general adult population that are able to exercise.

In terms of bodyweight exercise feasibility, all subjects were able to perform the balance exercises, which may support the early detection of various vestibular disorders, which affect balance and equilibrium, and increase with age following the 4th decade (50). The OLS used in this research has been shown to be highly predictive of all cause mortality in populations susceptible to balance deficits (4), and can be leveraged in combination with balance strengthening exercises to screen for changes to functional balance. The most difficult exercise in this study was the push up, especially for older subjects. This is unsurprising, given declines in muscle strength and mass with age, starting in the third decade of life (51). For individuals unable to perform bodyweight push ups, there is value in incrementally increasing upper body strength and endurance until push ups can be performed, since push up capacity is correlated with cardiovascular mortality (52). The majority of subjects were able to perform all lower body exercises. Lower extremity muscle mass and strength decline with age starting in the third decade, with muscle strength declining prior to overt changes to muscle mass (38). The core exercises used in this study were highly accessible, and are associated with foundational movements. Recently sit ups have fallen out of favor since research suggests that sit ups are not sensitive enough to assess core strength and endurance, and may lead to lower back or neck injury (36). Rather, crunches or plank holds are more commonly recommended for assessing core capacity (53, 54). The flexibility assessments were also well tolerated by the subjects in this study. Flexibility exercises are considered important for maintaining limb and joint range of motion along with mobility, and physical activity programs commonly include stretching exercises (29). In particular, lower body flexibility has been shown to be a predictor of lower back pain (55). Taken together, these exercises represent a highly accessible set of tools for assessment of strength, endurance, and movement factors associated with functional capacity and health.

The novel CRF assessments were also highly accessible, with the majority of subjects preferring the run in place to the burpee protocol. While the most common way to estimate CRF is through the use of cardiorespiratory responses to incremental increases in difficulty via submaximal tests (56), simpler heart rate recovery tests, such as the YMCA step test, have also shown strong correlation with VO_2_max (25). Besides stepping onto an inclined plane, other forms of repetitive movements, such as burpees (57), jumping jacks, or running in place (30, 57), cause similar changes to cardiorespiratory status as the step test (58), and are recommended for CRF assessments when additional equipment is not available. Both the running in place test and the burpee test provide a reliable estimate of VO_2_max which may be computed given age, sex, weight, and heart rate following exercise. Using VO_2_max estimates obtained from the step test as ground truth data, a linear regression was performed to estimate the coefficients for the various parameters. The mean absolute error averaged over 5 validation folds was found to be 1.24 ml/kg*min for the running in place test, and 1.33 ml/kg*min for the burpees test.

The reference measures correlated well with bodyweight exercise measures for upper body, lower body, and core capacity, along with flexibility and CRF. Increasing BMI was associated with higher leg extension and grip strength, and lower plank performance. Upper body capacity was also associated with core and lower body capacity, and flexibility, suggesting a clustering of fitness factors across body systems. Balance and flexibility were also positively correlated. Balance measure correlation was not completed given the high performance in the 3 stage balance test. For the study sample, increasing age was negatively associated with core, cardiorespiratory, and lower body capacity. This was expected since performance of these exercises has been shown to decrease with age. For instance, the 1 min OLS is expected to decline with age, starting in the 5th decade (4), and knee extension strength is also expected to decline starting in the third decade (38). Previous research has demonstrated high correlations between maximum sit ups and lower body measures such as maximum leg press (7, 59), upper body measures such as grip strength (7), and cardiorespiratory fitness measures such as VO_2_max (7). Correlation analyses from the current study showed that many of the strength and endurance features were significantly correlated, regardless of domain. That may be unsurprising in a relatively physically fit study sample, which may be following guidelines to perform aerobic activity and full-body muscle strengthening activities several times a week (60). Previously reported correlations between planks and sit ups (e.g., *r* = 0.43 for younger subjects) (61), were not observed in this sample, likely due to the plank ceiling effect. These results, combined with the duration of the test and the fact that holding a static plank is not associated with real-world activities of daily living, suggests that plank hold may not be appropriate for an active assessment. Rather, crunches, used in this study as a reference, represent a valid exercise candidate with real-world applicability and should be leveraged in an active assessment.

Myriad sex effects were also observed in the study sample. With the exception of abdominal capacity assessed using crunches, male subjects had higher strength and cardiorespiratory scores, and female subjects had higher flexibility and balance scores. The disparity in push up methods between the sexes has been criticized in the literature (22). Notably, the average load difference between modified and standard push ups is substantially lower than the average strength gap between the sexes (62). One group noted a high correlation between maximal modified and standard push ups, and promoted the use of standard push ups in female subjects, especially younger athletes (22). Based on this data, the current study provided female subjects with the option to perform either standard or modified push ups, leveraging recently published normative data for female standard push ups (22).

This study has several limitations. The convenience study sample may not be representative of the general population, limiting the generalizability of the study. The PAR-Q screened for contraindications, but does not address modifications for additional at-risk populations, such as those experiencing lower back strain. Some of the bodyweight exercises in this study were associated with a ceiling effect, including the squat plank holds, which limit their ability to correlate to reference measures. In addition, available data suggests that individuals who exercise most days each week will be able to hold the plank for an average of approximately 90 s (20) mean data from each demographic exceeded that in the current study, suggesting either a fitter than average population in this study or other differences in methodology. The impact of coaching or socioevaluative stress during the study may have caused subjects to maintain the static posture exercises for longer than previously reported (63). Such factors could influence performance and were not systematically measured or controlled beyond general encouragement. In addition, only 10% of subjects completed a maximal CRF test, which represents another limitation of the current study. As such, data from the run in place protocol was converted to VO_2_max values using reported equations from the step test rather than direct correlation with maximal testing (26). The portable fixed dynamometry approach leveraged in the current study is less accurate than gold standard isokinetic dynamometry. Follow-up analyses comparing the two approaches using an isometric knee extension protocol yielded a significant correlation coefficient (*r* = 0.96, *p* < 0.001 *n* = 11). In addition, lifestyle factors that have an impact on physical performance were not captured in the current study.

In summary, this study demonstrates the feasibility of using equipment-free, bodyweight exercises for a comprehensive fitness assessment in an adult population that is able to exercise. The proposed assessment battery offers a practical, equipment-free approach to measuring key fitness components that correlate with reference measures. The novel CRF measure provides promising alternatives to current instrumented approaches. This approach has the potential to facilitate early detection of fitness declines, guide personalized recommendations, and contribute to improved health and activity. Future research should focus on expanding to a general population including older adults who may exhibit more pronounced fitness declines, implementing longitudinal studies to validate these measures and explore their predictive value for long-term health outcomes, and providing integrations with existing programs or technology. For instance, assessment automation could be accomplished via the use of a smartphone camera, in conjunction with computer vision models that can identify exercises and count repetitions. Smartphone cameras represent a promising technology to support automatic implementation and scoring of a comprehensive exercise battery in a low cost, simple, and widely deployable manner. Recent work has shown the capability of computer vision models running on smartphones to accurately assess body composition (64) and functional movements (65). In addition, such an approach could be paired with individualized interventions, such as recommended changes to diet and activity, to improve longevity and healthspan. Such a smartphone-based system could offer a low cost, simple, and widely deployable solution to increase healthspan.

## Data Availability

The raw data supporting the conclusions of this article will be made available by the authors, without undue reservation.
